# Supplementary material for: NB-LRR Lineage-Specific Equipment Is Sorted Out by Sequence Pattern Adaptation and Domain Segment Shuffling
Source: Int J Mol Sci. 2022 Nov 17;23(22):14269. doi: 10.3390/ijms232214269 (PMC9696612; doi:10.3390/ijms232214269)
Supplement: Supplementary file 1 [file Figure S1.pdf › Figure S1.pdf]

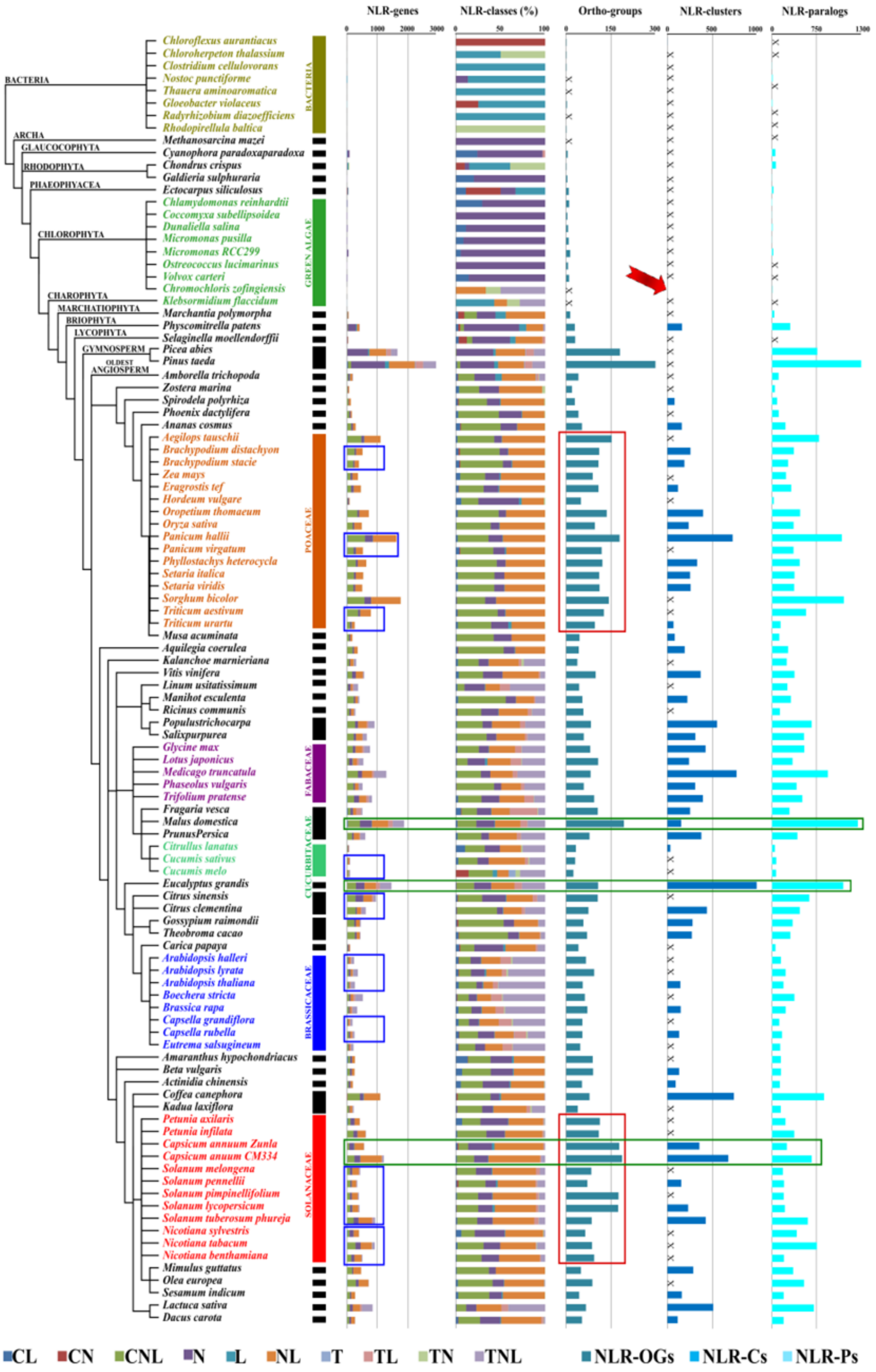

Figure S1. Arrangement and composition of NB-LRR genes in 104 genomes. The five bar charts, from left to right, represent for each genome the total number of NB-LRR genes (NLR-genes), the proportion of different NB-LRR protein classes (NLR-classes), the number of NB-LRR gene ortho-groups (NLR-OGs), NB-LRR clusters (NLR-Cs) and NB-LRR paralog genes (NLR-Ps). The colour boxes underlined the substantial differences of NB-LRR families. In the charts missing data are denoted with an "X" mark.
